# Supplementary material for: Macropinocytosis mediates neurotropism of Cryptococcus neoformans in a human organoid model of the blood-brain barrier
Source: Commun Biol. 2026 Jul 7;9:1430. doi: 10.1038/s42003-026-10597-y (PMC13720050; doi:10.1038/s42003-026-10597-y)
Supplement: Supplementary file 1 [file 42003_2026_10597_MOESM1_ESM.pdf]

nature portfolio

Peer Review File

Macropinocytosis mediates neurotropism of Cryptococcus neoformans in a human organoid model of the blood-brain barrier.

Corresponding Author: Dr Angie Gelli

This file contains all reviewer reports in order by version, followed by all author rebuttals in order by version.

Version 0:

Reviewer comments:

Reviewer #1

(Remarks to the Author)
In the manuscript entitled "Macropinocytosis mediates neurotropism of Cryptococcus neoformans in a human organoid model of the blood-brain barrier," the authors developed a 3D human blood-brain barrier (BBB) organoid model and, for the first time, demonstrated macropinocytosis as a potential mechanism for Cryptococcus neoformans to cross the BBB. The study also explored the roles of EphA2 and CD44 in this process. This study presents a novel concept and explores the mechanism in depth; however, some conclusions are undermined by a lack of logical rigor and insufficient experimental validation. Specific comments are provided below.

Major Comments:

1. The core claim that macropinocytosis serves as the primary mechanism for Cn internalization is not sufficiently substantiated by the presented evidence. This conclusion relies heavily on the use of amiloride (AML), which lacks specificity for macropinocytosis and may affect other cellular processes—such as Na^+/H^+ exchange. It is recommended that the authors employ additional macropinocytosis inhibitors to strengthen their conclusion. Furthermore, providing direct visual evidence—for example, Scanning Electron Microscopy (SEM) images capturing Cn within characteristic macropinocytic cups—would significantly reinforce the findings.

2. The authors observed reduced Cn internalization following EphA2 knockout and increased internalization with EphA2 overexpression. However, since prior studies have established a role for EphA2 in maintaining blood-brain barrier (BBB) integrity (Zhao et al. Neurosci Lett. 2020), the possibility that EphA2 influences Cn internalization through affecting the BBB integrity cannot be ruled out. The authors' statement in Lines 22–24 of Page 6, " This also further demonstrated that the lack of EphA2 may not have compromised the integrity of the organoid endothelium to the extent that it permitted Cn crossing.", is not substantiated by experimental evidence and remains speculative.

3. The evidence supporting the formation of EphA2-CD44 complex is insufficient. The AlphaFold3-predicted interface for this interaction has very low confidence (iPTM=0.11/0.13), a limitation the authors acknowledge due to the lack of prior structural data. Furthermore, the subsequent in-situ fluorescence-based proximity ligation assay (PLA), which indicates that the two proteins are within 40nm, is inadequate to confirm a direct protein-protein complex. The Discussion should be supplemented with a section addressing the limitations in the evidence for EphA2-CD44 complex formation and its direct link to macropinocytosis.

Minor Comments:

1. Several figures (Fugure 1b, 1c, 1f, 1g, 2a, 2b, 2c, 4a, 5g) are missing scale bars.

2. The placement of figure labels is also inconsistent throughout the manuscript, sometimes appearing inside and other times outside the figure panels, which disrupts the reader's flow and should be standardized for clarity.

Reviewer #2

(Remarks to the Author)
The authors studied the interaction between endothelial cells and Cn in an organoid model of the BBB.

The introduction is well written and clear.

The type of strain of the H99 lineage should be precise (H99 O, H99 C, …). A specific paragraph on yeasts strains used in this study should be
Why organoides are produced with immortalized brain endothelial cells OR primary bain endothelial cells ?
The result of the experiments could be affected by the type of cell put in the system.
No mention of the Fig 1B in the second result paragraph.
The figures should be organized by apperance in the text. 1b before 1c.
Page 4 line 19-20 : Precise the concentration of Cn exposed to orgabnoides
Fig 2a 2b : magnification is too low to really appreciate that Cn is close/in contact with the astrocytes or pericites. PAthology of these organoides should be possible to better observed the location of Cn cells.
Why deuterogattii or Saccharomyces have been used in parallel to Cn strain ?
Page 4 line 33 and 5 line 1 and 2 : this should be inserted in the discussion.
What is known about amiloride regarding other effects on cellular physiology outside macropinicytosis inhibition ? and on fungi outside vioability (growth capacities) ? (known to affect bacterial physiology)
Fig 2G in far from being precise in looking at intermediate effects on yeasts. (growth curved sould be performed and virulence factors checks)
Why not using other actin inhibitors to validate the effet of amiloride
Is the viability of the organoide the same with amiloride than without (2 wks) any toxic effect on the different cell types ?
Fig 3 : It is not clear if H99 was used with antibodies or with complemented serum to promote or stimulate phagocytosis.
Could be interesting to look at acapsular mutant which are internalyzed easily without opsonines and see if dextran is uptaken the same way .
Does serum limitation induce only macropinocytosis ? or other mechanisms of internalization could be on in addition ?

Page 6 line 10 : the work contingent whuld be replaced to increase clarity.
What type of protein is the Red Fluorescence Protein (RFP) ? why it localized at the periphery of the cell ? Itis not clear if parto of the EphA2 is present and added a florecent residue or other contructs ?

Page 6 19-20 ; The authors should explain how overexpression of EphA2 was perfomed

Page 6 line 29-33 Page 7 line 1-2. This is an assumption that should be discarded and potentially better put in perspectives in the discussion.

Explain better the possible connections between EPHA2 and cdc42. Why the authors checked cdc42 and not other small Rho GTPases ot other enzymes/proteins.
In what background CPS1 deficient mutant was generated ?
Interaction between CD44 and EPH2A rely only on structure predicting model. This should be validated biologically before proposing this model. A third party protein could play the role of intermeciary signal between those two.
C deuterogattii is also producing hyaluronic acid interacting with CD44. All the experiments done with H99 whould be donf with Cd in parallele including KO mutant, experiments on Figure 5A 5b or 4 a to d.

Reviewer #3

(Remarks to the Author)
This study presents an interesting and well-designed investigation showing that Cryptococcus neoformans induces EphA2 activation in endothelial cells, thereby triggering macropinocytosis and promoting its transcytosis across the blood–brain barrier (BBB). This process involves the activation of Rho-GTPase signaling through the interaction between the fungus and endothelial EphA2, and the authors highlight that the fungal surface component hyaluronic acid plays a key role in this signaling activation. Overall, the experimental design is compelling and the results are convincing, although several aspects of data presentation and explanation could be made clearer.

Page 3, line 12: First, the Introduction lacks sufficient detail regarding the physiological role of EphA2 and its function in brain endothelial cells. EphA2 can be phosphorylated through both ligand-dependent and ligand-independent mechanisms and is known to activate signaling pathways such as MAPK, Rho-GTPase, and PI3K/Akt, leading to cytoskeletal remodeling and modulation of cell–cell adhesion through integrins and cadherins. Since this paper focuses on the mechanism of action, briefly describing these fundamental aspects would help readers better understand the subsequent explanation of how the EphA2–CD44 axis mediates macropinocytosis.

Moreover, EphA2 activation has also been reported in infections by pathogen; adding a short statement about these examples would emphasize its broader relevance in host–pathogen interactions.

Regarding lines 15–18, where EphA2 recruitment of CD44 is mentioned, a concise description of the process—showing that CD44–hyaluronic acid binding leads to EphA2 transactivation and downstream Cdc42 signaling—would help readers grasp the mechanistic link more easily.

The results shown in Figure 1b appear to correspond to lines 16–19 (page 4). Either the text should explain these results earlier, or the order of Figures 1b and 1c should be reversed for a more logical flow. On page 4, lines 13–15, it would be useful to add a short background statement that endothelial cells typically restrict the passage of macromolecules above a certain size threshold, and that this experiment was performed to verify tight-junction integrity. The term “high inoculum” in line 20 is somewhat vague and should be replaced with the exact cell number used.

Throughout Figure 1, visual clarity could be improved. In figure 1b, including a DAPI single-channel image merged with Claudin-5 would clearly show nuclear and tight-junction localization. In figure 1d and 1e, different arrow colors should be used to distinguish between ruffled and flat regions. In figure 1f and 1g, the text labels (“Cn” and “Claudin-5”) are difficult to read; if figure 1g is a merged image, please indicate this explicitly and specify the color representation in the legend.

In page 4, line 32 to page 5, line 2, the interpretation that residual fluorescent signals represent capsule debris bound by antibody is reasonable. However, to confirm that this signal does not originate from unbound antibody remaining within the BBB interior, an antibody-only control would be necessary. It would be helpful to clarify whether a similar fluorescence pattern appears when antibodies are incubated alone; this control could be mentioned in the figure legend or supplementary materials.

For Figure 2, please provide detailed conditions for antibody incubation with C. neoformans, as well as the parameters for co-incubation with organoids and the washing steps (number of washes, buffer composition, etc.). These details should also be explicitly included in the Methods section. Although the quantification of internalized fungi is described in the Methods, if the calculation was based on the fluorescence intensity of capsule-bound antibody, it is necessary to verify that C. neoformans, C. deuterogattii, and S. cerevisiae bind the antibody with similar efficiency. Because S. cerevisiae may have weaker antibody binding, its lower fluorescence could be misinterpreted as reduced internalization. Some explanation or correction for these potential differences in binding efficiency would be important.

The results described in page 5, lines 11–12 would flow better if Figures 2f and 2g were switched. In lines 17–18, where amiloride (AML) is mentioned as being added before C. neoformans infection, the exact timing (e.g., 30 min or 1 h prior to inoculation) should be specified in the text or figure legend. In lines 21–23, it should be clearly stated that transferrin is transported by receptor-mediated endocytosis, not by macropinocytosis. This experiment serves as an important negative control, showing that amiloride specifically inhibits macropinocytosis rather than nonspecifically blocking all uptake processes. Expanding this explanation with a brief background description would make the result easier for readers to interpret.

In page 6, lines 5–7, and in Figure 3e, the different colors and shapes of data points should be defined in the legend. On page 8, line 17, the figure reference should be corrected from Figure 5c to Figure 6c. The results described in page 8, lines 28–32 appear more appropriate for Figure 6; additionally, only Figure 5g exists, while Figures 5h and 5e are missing, which should be corrected or clarified.

In the Discussion, it would be helpful to clearly state which of the three major BBB-crossing models—paracellular passage, Trojan-horse mechanism, or transcytosis—this study supports. Since the presented data strongly favor transcytosis, particularly macropinocytosis-based entry, explicitly emphasizing this and comparing it with previous models would strengthen the discussion.

Unlike the 2D transwell model, where TEER can be measured to assess barrier integrity directly, the 3D system lacks such quantitative readouts. If C. neoformans secretes factors such as GXM or lytic enzymes that cause subtle, undetectable damage to the barrier, these effects would not be visible in the 3D structure. Such minor damage could lead to an apparent increase in nonspecific internalization. A brief acknowledgment of this limitation would make the discussion more balanced and complete.

In page 17, line 7, the Western blot section states that a C. neoformans lysate was prepared, yet the figure appears to analyze endothelial-cell lysate. The authors should clarify which lysate was actually used and ensure consistency between the Methods and Results descriptions.

Reviewer #4

(Remarks to the Author)
I co-reviewed this manuscript with one of the reviewers who provided the listed reports. This is part of the Communications Biology initiative to facilitate training in peer review and to provide appropriate recognition for Early Career Researchers who co-review manuscripts.

Version 1:

Reviewer comments:

Reviewer #1

(Remarks to the Author)
The authors have adequately addressed my concerns, especially by providing new co-IP data for the EphA2–CD44 interaction and clarifying the BBB integrity issue. The totality of evidence (amiloride inhibition, FITC-Dextran uptake, Cdc42 activation, and prior published data) supports their conclusion. All minor issues have been corrected. I therefore recommend acceptance of the manuscript.

Reviewer #2

(Remarks to the Author)

Reviewer #3

(Remarks to the Author)
I have carefully reviewed the revised manuscript and the authors' responses to the reviewers' comments. The authors have addressed the major concerns appropriately, and the manuscript has been significantly improved following revision. I believe the study is now suitable for publication in its current form.

Reviewer #4

(Remarks to the Author)
I co-reviewed this manuscript with one of the reviewers who provided the listed reports. This is part of the Communications Biology initiative to facilitate training in peer review and to provide appropriate recognition for Early Career Researchers who co-review manuscripts.

Open Access This Peer Review File is licensed under a Creative Commons Attribution 4.0 International License, which permits use, sharing, adaptation, distribution and reproduction in any medium or format, as long as you give appropriate credit to the original author(s) and the source, provide a link to the Creative Commons license, and indicate if changes were made.
In cases where reviewers are anonymous, credit should be given to 'Anonymous Referee' and the source.
The images or other third party material in this Peer Review File are included in the article's Creative Commons license, unless indicated otherwise in a credit line to the material. If material is not included in the article's Creative Commons license and your intended use is not permitted by statutory regulation or exceeds the permitted use, you will need to obtain permission directly from the copyright holder.
To view a copy of this license, visit https://creativecommons.org/licenses/by/4.0/

We are grateful to the reviewer's for their many insightful comments and suggestions. Below we have addressed each comment with either new experimental data, and/or clarifications/additions to the manuscript. Please see below for our responses to all comments.

Reviewer #1 (Remarks to the Author):

In the manuscript entitled "Macropinocytosis mediates neurotropism of Cryptococcus neoformans in a human organoid model of the blood-brain barrier," the authors developed a 3D human blood-brain barrier (BBB) organoid model and, for the first time, demonstrated macropinocytosis as a potential mechanism for Cryptococcus neoformans to cross the BBB. The study also explored the roles of EphA2 and CD44 in this process. This study presents a novel concept and explores the mechanism in depth; however, some conclusions are undermined by a lack of logical rigor and insufficient experimental validation. Specific comments are provided below.

Major Comments:

1. The core claim that macropinocytosis serves as the primary mechanism for Cn internalization is not sufficiently substantiated by the presented evidence. This conclusion relies heavily on the use of amiloride (AML), which lacks specificity for macropinocytosis and may affect other cellular processes—such as Na^+/H^+ exchange. It is recommended that the authors employ additional macropinocytosis inhibitors to strengthen their conclusion. Furthermore, providing direct visual evidence—for example, Scanning Electron Microscopy (SEM) images capturing Cn within characteristic macropinocytic cups—would significantly reinforce the findings.

Our conclusion that macropinocytosis is involved in *Cn* entry is not based solely on the inhibitory effects of amiloride. We have also demonstrated that the fluid phase marker, FITC-Dextran (a classic indicator of macropinocytosis) was engulfed only when brain endothelial cells were exposed to *Cn*, similar to that observed during serum starvation (a well-known inducer of macropinocytosis) in non-exposed cells; and we demonstrated the recruitment and activation of Cdc42, a Rho-GTPase and a crucial regulator of macropinocytosis. These three lines of evidence (amiloride inhibition, FITC-Dextran uptake and Cdc42 activation), in addition to our previous published study where we identified Phalloidin-stained cup-formations in brain endothelial cells surrounding *Cn* (please see below; *Aaron et al., 2018 Cell Micro*), strongly support macropinocytosis as the mechanism of entry.

As for testing other inhibitors of macropinocytosis, other than amiloride and a derivative of amiloride (EIPA), there are no specific inhibitors of macropinocytosis. There are more general inhibitors of membrane dynamics or actin polymerization, like cytochalasin D, which has been previously reported as blocking *Cn* crossing of brain endothelial cells.

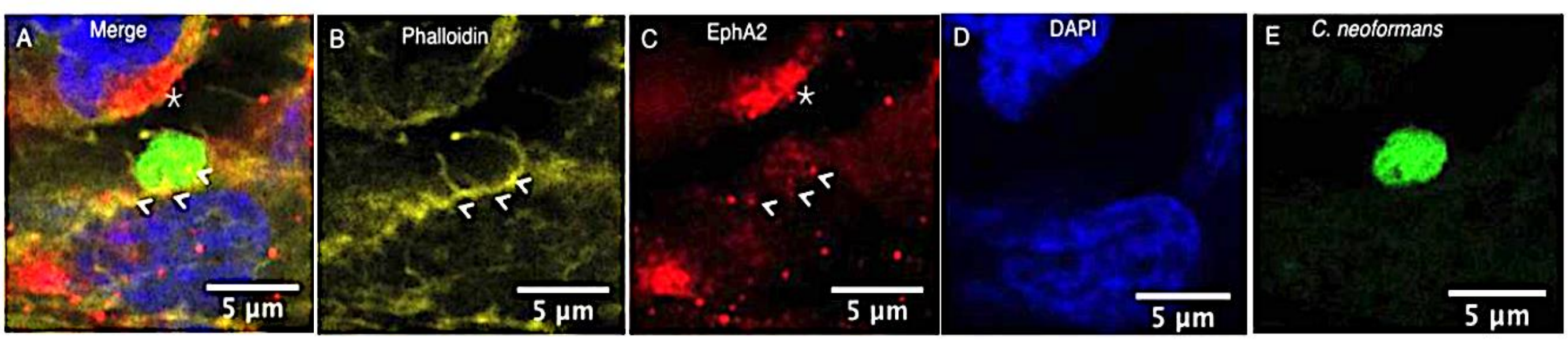

2. The authors observed reduced Cn internalization following EphA2 knockout and increased internalization with EphA2 overexpression. However, since prior studies have established a role for EphA2 in maintaining blood-brain barrier (BBB) integrity (Zhao et al. Neurosci Lett. 2020), the possibility that EphA2 influences Cn internalization through affecting the BBB integrity cannot be ruled out. The authors' statement in Lines 22–24 of Page 6, " This also further demonstrated that the lack of EphA2 may not have compromised the integrity of the organoid endothelium to the extent that it permitted Cn crossing.", is not substantiated by experimental evidence and remains speculative.

EphA2 is not *required* for the integrity of the BBB per se, rather studies have demonstrated that EphA2 activation/up-regulation can increase the permeability of the barrier. Our data support the conclusion that *Cn*-induced activity of EphA2 facilitates the entry of *Cn* via an endocytic process. To further validate these results,

we included additional supplementary data which show that the localization of Claudin5, the major tight junction protein, is not significantly altered in EphA2 +/+ or EphA2 KO endothelial cells (whether exposed to *Cn* or not). The lack of requirement for EphA2 in maintaining the BBB integrity is also supported by data from our previous study (Aaron et al, 2018) showing that reduction of EphA2 expression level via RNAi or blocking EphA2 activity with either an antibody or with an inhibitor did not alter the TEERs or permeability to FITC-dextrans.

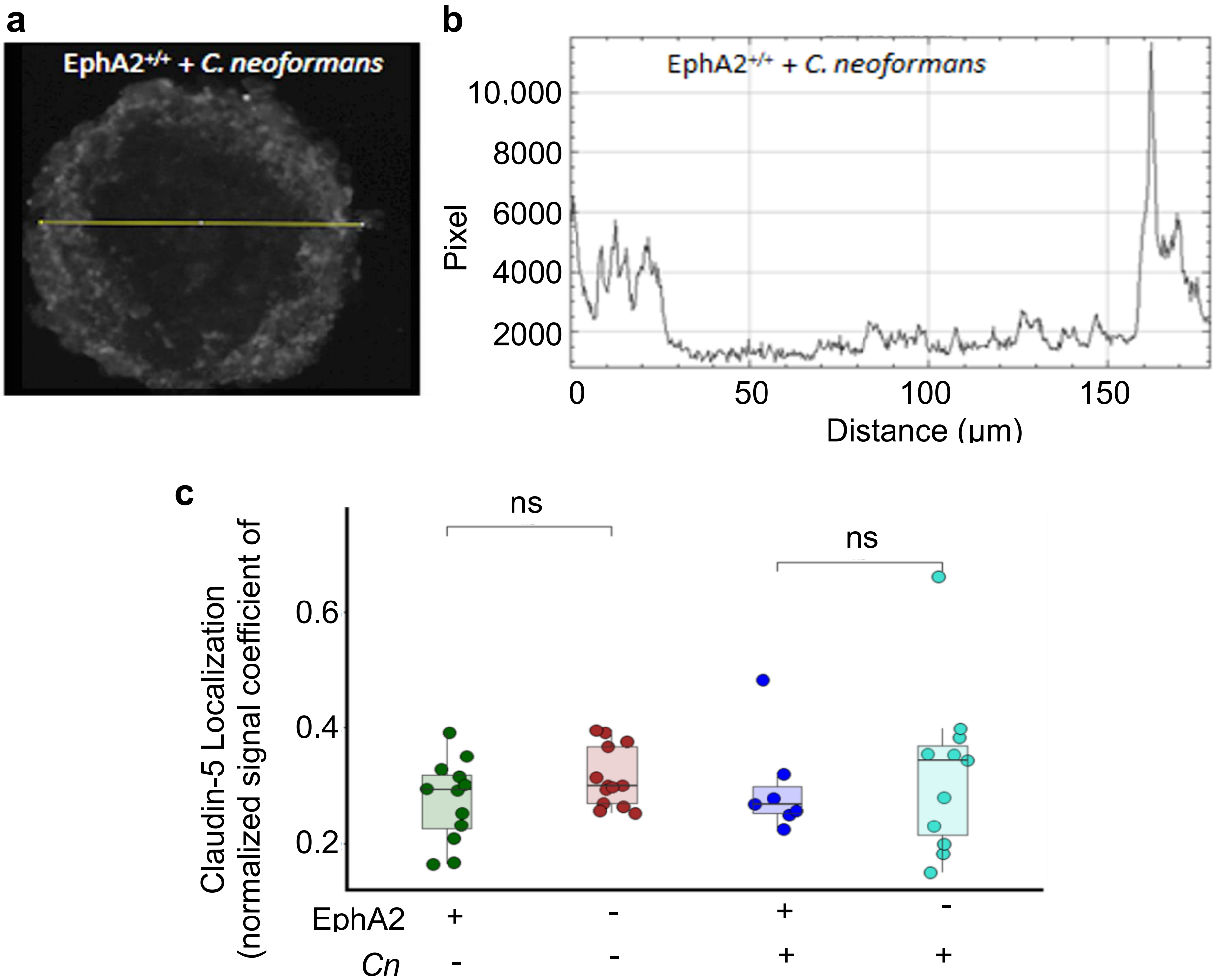

Supplementary Fig.6. Claudin5 expression in *EphA2* KO organoids exposed to *Cn* a) Claudin5 signal (Cy5 channel) in cross-sections of BBB organoids. Sections were probed with rabbit anti-claudin5 (1:1000, ab15106, Abcam) primary antibodies and goat anti-rabbit Cy5 (1:1000, ab6565, Abcam) secondary antibodies. **b)** Histograms corresponding to the pixel intensities measured across the diameter of organoids, prior to normalization and downstream analysis. **c)** Claudin5 localization in organoids from EphA2+ or *EphA2* KO endothelial cells in the presence and absence of *Cn* (24 h coincubation). To create a metric of claudin5 localization within organoids, pixel intensity in the claudin5 (Cy5) channel across the diameter of organoid sections was acquired in ImageJ using the Plot Profile tool, normalized to the maximum value, and the coefficient of variation for each normalized vector was then taken. Points represent the average coefficient of variation across multiple sections for individual organoids. Data drawn from three independent replicates. There is no significant difference between EphA2+ and *EphA2* KO organoids

3. The evidence supporting the formation of EphA2-CD44 complex is insufficient. The AlphaFold3-predicted interface for this interaction has very low confidence (iPTM=0.11/0.13), a limitation the authors acknowledge due to the lack of prior structural data. Furthermore, the subsequent in-situ fluorescence-based proximity ligation assay (PLA), which indicates that the two proteins are within 40nm, is inadequate to confirm a direct protein-protein complex. The Discussion should be supplemented with a section addressing the limitations in the evidence for EphA2-CD44 complex formation and its direct link to macropinocytosis.

Yes, we agree with the reviewer that the PLG assay is not a demonstration of a direct interaction between CD44 and EphA2. To address this limitation, we included new data from pull-down assays. $CD44^{FLAG}$ and/or $EphA2^{V5}$ were transiently expressed in HEK293T cells for 48 h at which point cells were exposed to *Cn* for 2 h and immediately lysed for co-immunoprecipitation (co-IP) studies. The co-IP studies identified CD44 as a binding partner of EphA2 consistent with the PLA data and the in-silico analysis. The data shown below are included in the manuscript in Fig. 5h. Images of the entire protein blots are included in Supp. Fig. 7.

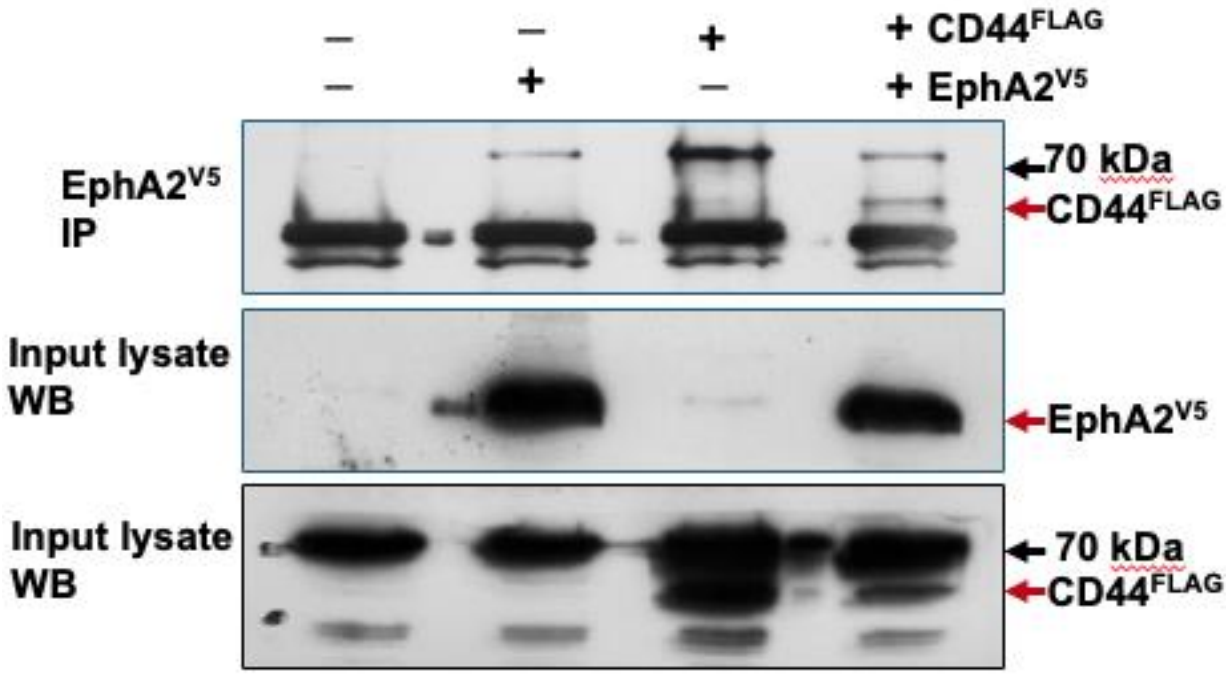

Minor Comments:

1. Several figures (Figure 1b, 1c, 1f, 1g, 2a, 2b, 2c, 4a, 5g) are missing scale bars.

This has been corrected.

2. The placement of figure labels is also inconsistent throughout the manuscript, sometimes appearing inside and other times outside the figure panels, which disrupts the reader's flow and should be standardized for clarity.

This has been corrected

Reviewer #2 (Remarks to the Author):

The authors studied the interaction between endothelial cells and Cn in an organoid model of the BBB.
The introduction is well written and clear.
We thank the reviewer.

The type of strain of the H99 lineage should be precise (H99 O, H99 C, …). A specific paragraph on yeasts strains used in this study should be
This has been corrected in the manuscript as shown below:
"Fungal strains used in this study are as follows: Cryptococcus neoformans (Cn) strain H99 was a human clinical isolate from a patient with Hodgkin's disease and was originally purchased from ATCC (ATCC 208821). Cryptococcus gattii (Cd) was also a human clinical isolate with the strain designation, NIH 444, and was obtained from ATCC (ATCC 32609). Saccharomyces cerevisiae (Sc) is a wild-type strain of the commonly known Baker's yeast. The cps1Δ hyaluronic-deficient strain is the KN99 lab strain lacking the CPS1 gene. The commonly used KN99 strain was derived from the H99 background."

Why organoides are produced with immortalized brain endothelial cells OR primary bain endothelial cells ?
Primary brain microvascular endothelial cells (BMECs) were used as a comparison to illustrate the point that both immortalized and primary BMECs were phenotypically and functionally similar. Since we used the immortalized cell line, HCMEC/D3, to make the *EphA2* KO, it was important to validate this point. Immortalized BMECs are used in all organoid-based experiments except those shown in Fig. 1f and 1g. The appropriateness of this immortalized line to investigate BBB crossing by *Cn* has been repeatedly demonstrated, such as the work done in Vu et al. (2009) and Weksler et al. (2013).

No mention of the Fig 1B in the second result paragraph.
The figures should be organized by apperance in the text. 1b before 1c.
This had been corrected in the manuscript.

Page 4 line 19-20 : Precise the concentration of Cn exposed to orgabnoides
The concentration is 2 x 10^6 cells per well or 2 x 10^7 cells/mL - consistent for all organoid experiments.

Fig 2a 2b : magnification is too low to really appreciate that Cn is close/in contact with the astrocytes or pericites. PAthology of these organoides should be possible to better observed the location of Cn cells.
We now provide high-resolution images of each of the channels, along with updated scale bars. While we concede that the interaction between pericytes and astrocytes cannot be fully characterized from this figure alone, we respectfully disagree that we cannot tell that these cells and *Cn* are in close contact. *Cn* are deeply internalized inside of the organoids and in contact with cells that do not express Claudin5, which, by process of elimination, must be astrocytes and pericytes. Additionally, direct interactions between *Cn* and cells of the brain parenchyma are well documented *in vivo* (Woo and Martinez 2021; Hamed et al. 2023).

Why deuterogattii or Saccharomyces have been used in parallel to Cn strain ?
These strains were used for validating the organoid model rather than for directly investigating macropinocytosis. Since we were developing a new model, it was important to validate it by testing other strains and recapitulating clinical results before applying the 3D BBB organoids to investigate macropinocytosis. A similar approach was taken in a recent article that developed a “neurovascular-unit-on-chip” model, (Kim, et al., 2021) in which *C. gattii* was used as a point of comparison to *Cn. Saccharomyces cerevisiae* was straightforwardly used as a negative control for false crossing incidence, as this species is not expected to cross an intact model BBB.

Page 4 line 33 and 5 line 1 and 2 : this should be inserted in the discussion.
This has been corrected as suggested.

What is known about amiloride regarding other effects on cellular physiology outside macropinicytosis inhibition ? and on fungi outside vioability (growth capacities) ? (known to affect bacterial physiology)
We are confident based on a previous publication that at the concentration of amiloride used in the current study does not affect the viability/function of *Cn*. At the concentration used for macropinocytosis inhibition, amiloride had negligible impact on the expression of two key virulence traits – growth at 37ºC and production of the polysaccharide capsule. We found no statistical difference in capsule thickness between *Cn* treated with vehicle (DMSO) or *Cn* treated with amiloride under both cell-culture growth condition or capsule-inducing condition (see Fig. 2g and Supp. Fig. 4).

Fig 2G in far from being precise in looking at intermediate effects on yeasts. (growth curved sould be performed and virulence factors checks)
We have confirmed that the concentration of amiloride used in our study (250 µM) does not affect the viability of *Cn* under the same conditions used in the 2D transwell and 3D organoid model. This is illustrated by the spot sensitivity assay (Fig. 2f), which is routinely used to assess fungal viability. At a higher, toxic concentration of amiloride, one publication reported alterations in calcium homeostasis and apoptosis due to depleted ER stores. To pick a particularly relevant virulence factor, we found no statistical difference in capsule thickness between *Cn* treated with vehicle (DMSO) or *Cn* treated with amiloride under both cell-culture growth condition or capsule-inducing condition (see Fig. 2g and Supp. Fig. 4).

The capsule data is also a critical control for potential defects in BBB crossing of *Cn* due to amiloride treatment because the capsule contains hyaluronic acid which is required for binding of *Cn* to the CD44 receptor on brain endothelial cells. The lack of a significant difference in capsule thickness of *Cn* under amiloride treatment and control growth conditions not only attests to the fact that amiloride treatment at the working concentration (250 µM) did not change the expression of this major virulence factor, amiloride treatment also did not preclude *Cn* from binding to CD44 and crossing the BBB. Thus, we are confident that the activity of amiloride acted solely on brain endothelial cells by inhibiting macropinocytosis.

Why not using other actin inhibitors to validate the effet of amiloride
The inhibitor of actin polymerization, cytochalasin D, was previously reported (Jong et al., 2008, Cell Microbiol) to block *Cn* crossing of brain endothelial cells.

Is the viability of the organoide the same with amiloride than without (2 wks) any toxic effect on the different cell types ?

The organoids were only briefly exposed to amiloride relative to the total amount of time they were maintained. Additionally, any toxic effect on the organoids themselves would tend to promote measured crossing, which was not observed.

Fig 3 : It is not clear if H99 was used with antibodies or with complemented serum to promote or stimulate phagocytosis.
Exposure of BMECs with *Cn* **alone** is enough to induce macropinocytosis promoting the uptake of FITC-Dextran. In other words, neither antibodies nor complement serum were used in the presence of *Cn* to stimulate the uptake of FITC-Dextran via macropinocytosis. Antibodies or complement are not required for the uptake of *Cn* by endothelial cells. The process we are investigating is not phagocytosis-dependent.

Could be interesting to look at acapsular mutant which are internalyzed easily without opsonines and see if dextran is uptaken the same way .
Brain endothelial cells do not behave like macrophages, therefore we would expect that opsonization would not have any effect on *Cn* internalization by brain endothelial cells Unlike other forms of endocytosis, macropinocytosis is typically stimulated by epidermal growth factors or tyrosine kinase receptors that induce a global change in actin dynamics within a cell, leading to the nonspecific uptake of extracellular fluid/cargo that vary greatly in size.

Does serum limitation induce only macropinocytosis ? or other mechanisms of internalization could be on in addition ?
Serum starvation induces macropinocytosis. Cells are known to take large gulps (macropinosomes) of their environment via macropinocytosis when starved of serum. Serum starvation can also induce clathrin-mediated endocytosis (which is cargo-specific), but this would not be the process involved in FITC-Dextran uptake due to the size of FITC-Dextran beads necessitating the large size of endosomes unique to macropinocytosis.

Page 6 line 10 : the work contingent whuld be replaced to increase clarity.
Contingent has been replaced with dependent.

What type of protein is the Red Fluorescence Protein (RFP) ? why it localized at the periphery of the cell ? Itis not clear if parto of the EphA2 is present and added a florecent residue or other contructs ?
We apologize for the confusion. The image shown is a section of a 3D organoid and the Red Fluorescence Protein (RFP) delineates the endothelial cell layer of the organoid rather than the periphery of an individual endothelial cell. RFP is a marker for CRISPR-Cas9-mediated *EphA2* KO that we introduced in brain endothelial cells. The expression of the RFP marker show that *EphA2* KO endothelial cells have the same peripheral localization within the 3D organoid as EphA2+ endothelial cells (see Figure 1, Supplementary Figure 2), confirming that knocking out EphA2 from BMECs did not result in alterations of the organoid endothelial cell layer. The expression of RFP also helps validate (based on the construction of the CRISPR-Cas9 plasmid) deletion of *EphA2* and western blot confirms the lack of EphA2 protein expression. We have added a scale bar to the image to clarify the scale of the organoid.

Page 6 19-20 ; The authors should explain how overexpression of EphA2 was perfomed
To generate iBMECs overexpressing EphA2, lentiviral particles were first produced by transient transfection of HEK293T cells using a third-generation packaging system. Briefly, HEK293T cells were co-transfected with the transfer plasmid (pLenti-) and an optimized mix of packaging plasmids - pLP1, pLP2, and pLP/VSVG (ViraPower Lentiviral packaging mix, thermofisher scientific) using Lipofectamine 2000 (Thermo Fisher Scientific) according to the manufacturer's instructions. Viral supernatants were collected 48 and 72 h post-transfection, filtered through a 0.45 µm PVDF filter, and stored at –80°C.
iBMECs were transduced with viral supernatant at an MOI of 5 and supplemented with 8 µg/mL polybrene (Sigma-Aldrich). After 48 h, the medium was replaced, and cells were allowed to recover. Transduced cells were selected using blasticidin (1.5 µg/mL) for 3 days before analyzing expression via western blot analysis (anti-V5 tag antibody, ab27671, abcam, dilution 1:1000).

Page 6 line 29-33 Page 7 line 1-2. This is an assumption that should be discarded and potentially better put in perspectives in the discussion.
This has been corrected as suggested.

Explain better the possible connections between EPHA2 and cdc42. Why the authors checked cdc42 and not other small Rho GTPases ot other enzymes/proteins.
We examined the activation of Rho GTPases during the process of *Cn* internalization because of their prominent role in macropinocytosis. Rho GTPases play a crucial role in mediating ruffle formation, macropinosome closure and membrane trafficking in macropinocytosis. We initially checked the activation of Rac1 and did not see a significant increase in GTPase-bound Rac1; however, we found a significant increase in GTP-bound Cdc42. This is consistent with the Chen group's finding that Cdc42 plays a significant role in the ruffling during *Salmonella typhimurium* invasion and macropinocytosis in immature dendritic cells.
Garrett, W. S., Chen, L. M., Kroschewski, R., Ebersold, M., Turley, S., Trombetta, S., ... & Mellman, I. (2000). Developmental control of endocytosis in dendritic cells by Cdc42. Cell, 102(3), 325-334; Chen, L. M., Hobbie, S., & Galan, J. E. (1996). Requirement of CDC42 for Salmonella-induced cytoskeletal and nuclear responses. Science, 274(5295), 2115-2118; Norbury, C. C. (2006). Drinking a lot is good for dendritic cells. Immunology, 117(4), 443-451.

In what background CPS1 deficient mutant was generated ?
*The cps1*Δ hyaluronic-deficient strain was generated in the KN99 background (Kwang-Woo et al., 2015) which is derived from the H99 strain that we used throughout the study.

Interaction between CD44 and EPH2A rely only on structure predicting model. This should be validated biologically before proposing this model. A third party protein could play the role of intermeciary signal between those two.
We have included new data (Fig. 5h) of a co-immunoprecipitation study that validates the CD44-EphA2 interaction.

C deuterogattii is also producing hyaluronic acid interacting with CD44. All the experiments done with H99 whould be donf with Cd in parallele including KO mutant, experiments on Figure 5A 5b or 4 a to d.
While we acknowledge that *C. deuterogattii (Cd)* also produces hyaluronic acid that can interact with CD44, we respectfully disagree that parallel experiments with both *Cn* and *Cd* are needed to demonstrate macropinocytosis as the primary mechanism used by *Cn* to cross the BBB. The reason for including *Cd* was to establish whether the 3D BBB organoid model could distinguish *Cn* from other fungal cells. While it would be interesting to perform further analyses with *Cd*, this would not inform or add to the intended aim of the study and is thus beyond the scope of the current study.

Reviewer #3 (Remarks to the Author):

This study presents an interesting and well-designed investigation showing that Cryptococcus neoformans induces EphA2 activation in endothelial cells, thereby triggering macropinocytosis and promoting its transcytosis across the blood–brain barrier (BBB). This process involves the activation of Rho-GTPase signaling through the interaction between the fungus and endothelial EphA2, and the authors highlight that the fungal surface component hyaluronic acid plays a key role in this signaling activation. Overall, the experimental design is compelling and the results are convincing, although several aspects of data presentation and explanation could be made clearer.

Page 3, line 12: First, the Introduction lacks sufficient detail regarding the physiological role of EphA2 and its function in brain endothelial cells. EphA2 can be phosphorylated through both ligand-dependent and ligand-independent mechanisms and is known to activate signaling pathways such as MAPK, Rho-GTPase, and PI3K/Akt, leading to cytoskeletal remodeling and modulation of cell–cell adhesion through integrins and cadherins. Since this paper focuses on the mechanism of action, briefly describing these fundamental aspects would help readers better understand the subsequent explanation of how the EphA2–CD44 axis mediates macropinocytosis.
Additional background information regarding EphA2 has been added to the introduction.

Moreover, EphA2 activation has also been reported in infections by pathogen; adding a short statement about these examples would emphasize its broader relevance in host–pathogen interactions.
This has been addressed in the discussion.

Regarding lines 15–18, where EphA2 recruitment of CD44 is mentioned, a concise description of the

process—showing that CD44–hyaluronic acid binding leads to EphA2 transactivation and downstream Cdc42 signaling—would help readers grasp the mechanistic link more easily.
We thank the reviewer for this suggestion. More detail has been added to the discussion.

The results shown in Figure 1b appear to correspond to lines 16–19 (page 4). Either the text should explain these results earlier, or the order of Figures 1b and 1c should be reversed for a more logical flow.
This has been addressed.

On page 4, lines 13–15, it would be useful to add a short background statement that endothelial cells typically restrict the passage of macromolecules above a certain size threshold, and that this experiment was performed to verify tight-junction integrity.
This has been added to the text. Typically brain endothelial cells exclude the passive entry of molecules larger than 600 Da (Kadry et al., 2020).

The term “high inoculum” in line 20 is somewhat vague and should be replaced with the exact cell number used.
This has been clarified (2 x 10^6 fungal cells/well or 2 x 10^7 fungal cells/mL) used throughout the study.

Throughout Figure 1, visual clarity could be improved. In figure 1b, including a DAPI single-channel image merged with Claudin-5 would clearly show nuclear and tight-junction localization. In figure 1d and 1e, different arrow colors should be used to distinguish between ruffled and flat regions. In figure 1f and 1g, the text labels (“Cn” and “Claudin-5”) are difficult to read; if figure 1g is a merged image, please indicate this explicitly and specify the color representation in the legend.
The figures have been updated along the lines suggested, with most of the fluorescence microscopy images now represented by their component channels in addition to the merged images, and these images now contain scale bars.

In page 4, line 32 to page 5, line 2, the interpretation that residual fluorescent signals represent capsule debris bound by antibody is reasonable. However, to confirm that this **signal does not originate from unbound antibody remaining within the BBB interior**, an antibody-only control would be necessary. It would be helpful to clarify whether a similar fluorescence pattern appears when antibodies are incubated alone; this control could be mentioned in the figure legend or supplementary materials.
Here is a composite image of a section of a BBB organoid exposed to both the claudin 5 (red) the 18B7 anti-capsule antibodies (green, no signal detected), plus DAPI, in the absence of *Cn*. Despite robust signal in the claudin5 channel localized around the organoid periphery, as expected, we did not detect puncta associated with the anti-capsule antibody in this control, suggesting that the staining in 2a-c is associated exclusively with *Cn* capsule components and not binding to mammalian ECM components, for example. The image below has been incorporated as supplementary figure S2 g- j. Note in i, which is the channel associated with the 18b7 anti-capsule antibody, the gain has been elevated to maximum settings, showing non-specific autofluorescence rather than the puncta characteristic of the sloughed capsule in fig 2a-c.

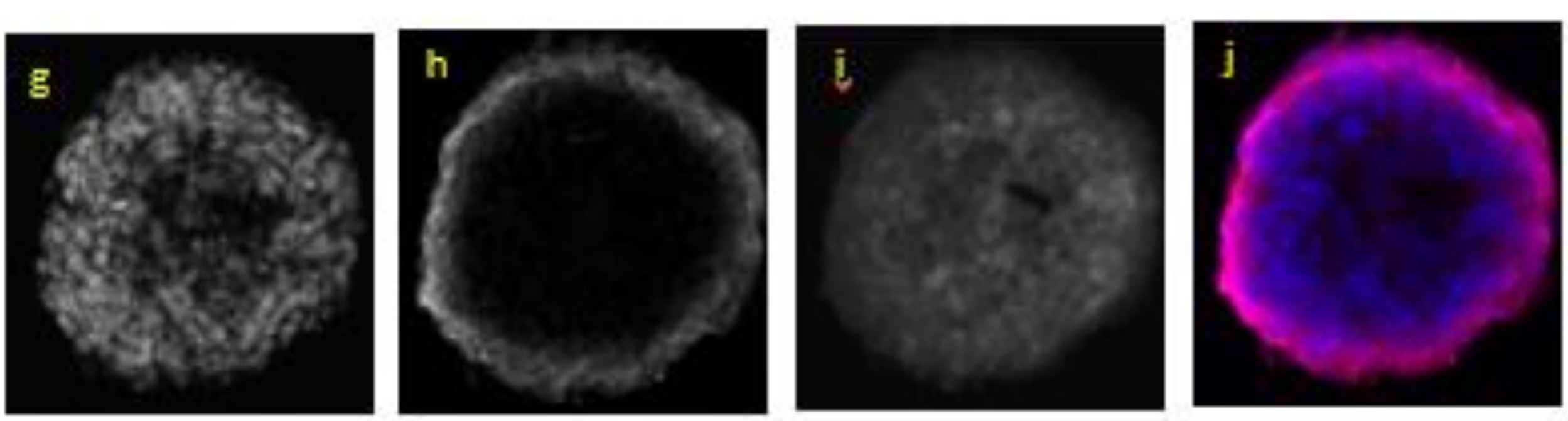

Additionally, in figure 2a-c, capsule antibodies were only applied after washing and fixing samples, and antibodies therefore did not “ride in” with live fungi. If the antibody staining were non-specific, we would see similar patterns between the claudin5 antibodies (red) and the capsular antibodies (green). This was not what we observed, as there is a clear difference between these two channels. We have split the channels apart to make this difference clearer.

For Figure 2, please provide detailed conditions for antibody incubation with C. neoformans, as well as the parameters for co-incubation with organoids and the washing steps (number of washes, buffer composition, etc.). These details should also be explicitly included in the Methods section. Although the quantification of internalized fungi is described in the Methods, if the calculation was based on the fluorescence intensity of capsule-bound antibody, it is necessary to verify that C. neoformans, C. deuterogattii, and S. cerevisiae bind the antibody with similar efficiency. Because S. cerevisiae may have weaker antibody binding, its lower fluorescence could be misinterpreted as reduced internalization. Some explanation or correction for these potential differences in binding efficiency would be important.

Antibodies were not used for quantifying differences in crossing between fungal strains. Rather, in experiments in which quantitative data was obtained on BBB crossing in organoids (specifically, figures 2d, 2e, and 4c, and not preliminary validation experiments as in 2a-c) fungi (*Cn, Cd, Sc*) were stained with CFSE, as detailed in the methods. Crossing was counted as discrete events and not overall fluorescence, and there the method of counting was straightforward: a border was automatically drawn around organoids using the DAPI channel, and all fungal cells (CFSE channel) internal to that were counted as internalized. We did verify that *Cn* and *Cd* were equally detectable after CFSE staining, using an ImageJ macro to compare particle counts in the green and transmitted light channels:

c

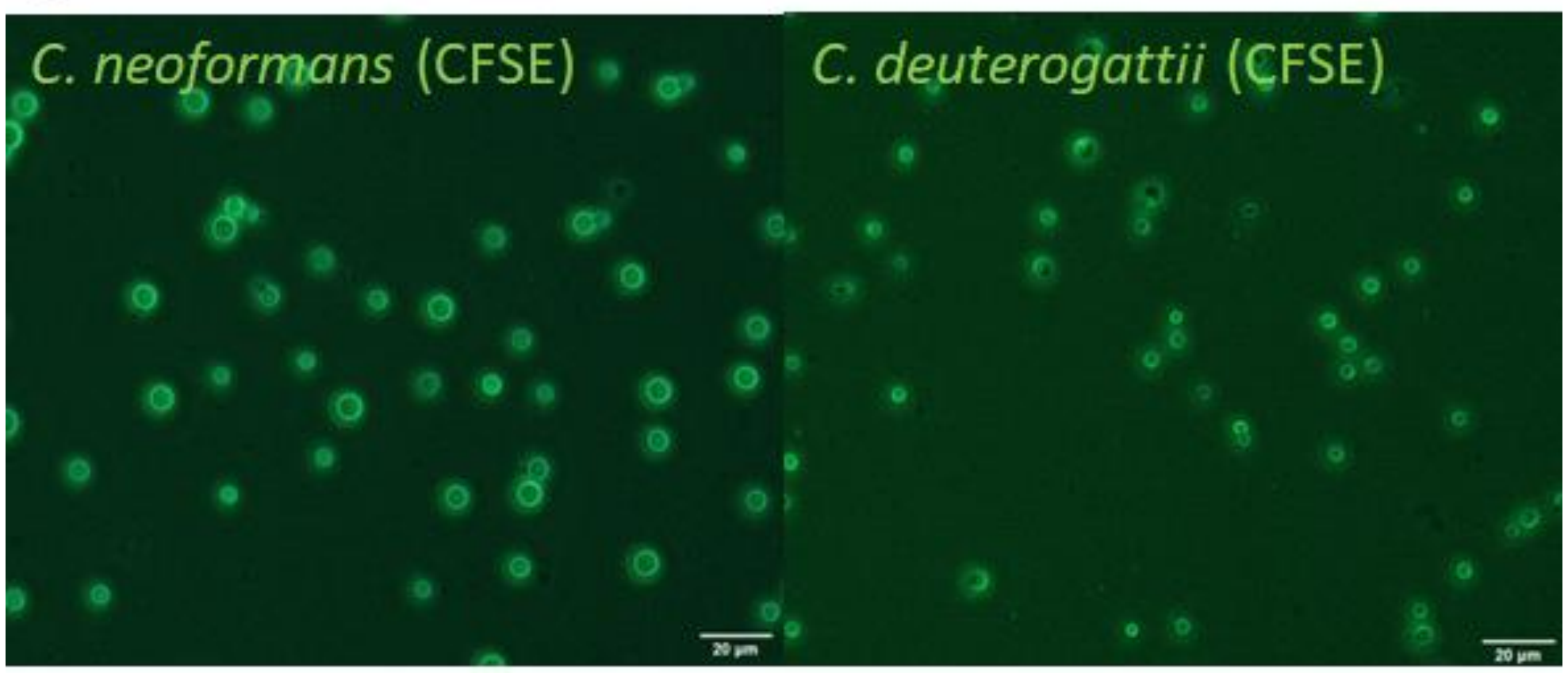

d

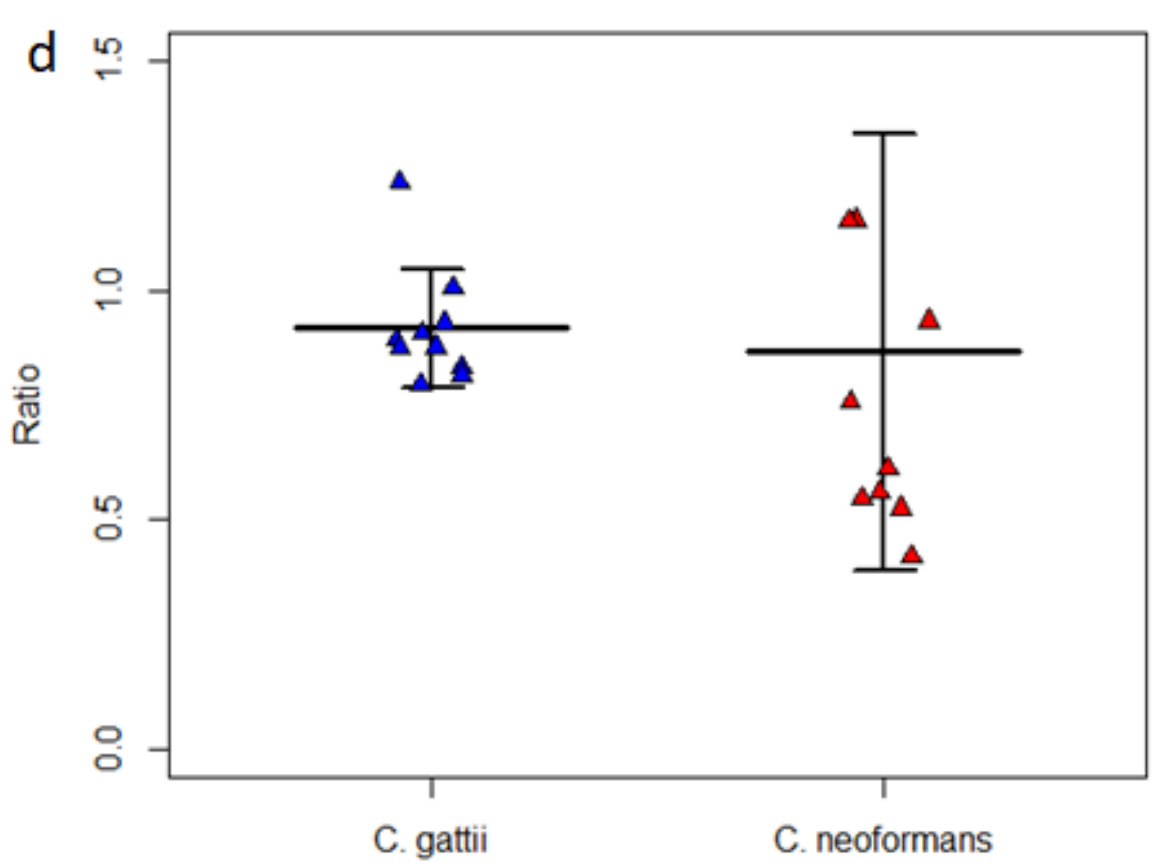

These figures, demonstrating similar uptake of CFSE, have been included in Supp. Fig. 3 as Fig. 3b and Fig. 3c.

The results described in page 5, lines 11–12 would flow better if Figures 2f and 2g were switched.

The new order of Figs 2f and 2g align well with the narrative.

In lines 17–18, where amiloride (AML) is mentioned as being added before C. neoformans infection, the exact timing (e.g., 30 min or 1 h prior to inoculation) should be specified in the text or figure legend.

This has been added.

In lines 21–23, it should be clearly stated that transferrin is transported by receptor-mediated endocytosis, not by macropinocytosis. This experiment serves as an important negative control, showing that amiloride specifically inhibits macropinocytosis rather than nonspecifically blocking all uptake processes. Expanding this explanation with a brief background description would make the result easier for readers to interpret.

We have included a sentence clarifying that transferrin is used a negative control for macropinocytosis because transferrin is known to cross the BBB by receptor-mediated and clathrin-dependent endocytosis.

In page 6, lines 5–7, and in Figure 3e, the different colors and shapes of data points should be defined in the legend.

This has been corrected.

On page 8, line 17, the figure reference should be corrected from Figure 5c to Figure 6c. The results described in page 8, lines 28–32 appear more appropriate for Figure 6; additionally, only Figure 5g exists, while Figures 5h and 5e are missing, which should be corrected or clarified.
This has been corrected.

In the Discussion, it would be helpful to clearly state which of the three major BBB-crossing models—paracellular passage, Trojan-horse mechanism, or transcytosis—this study supports. Since the presented data strongly favor transcytosis, particularly macropinocytosis-based entry, explicitly emphasizing this and comparing it with previous models would strengthen the discussion.
We have included this paragraph in the discussion: Although this study does not preclude either the Trojan-horse or pericellular routes, the mechanism described best supports the transcellular pathway. There is extensive evidence for transcellular crossing, both in *in vitro* (Chang et al. 2004) and *in vivo* (Shi et al. 2012; Davis et al. 2016). Some of this previous work firmly situates free *Cn* cells within the cytoplasm of endothelial cells during crossing (Chen et al. 2021; Li et al. 2021). These previous studies relied on a variety of methods to demonstrate transcellular crossing, including ultrastructural analysis of transwell models (Chang et al. 2004), intravital microscopy of brain blood vessels along with dyed *Cn* (Shi et al. 2012), and innovative flow-cytometry based protection assays capable of quantifying the relative proportion and importance of transcellular crossing events in a rodent model (Chen et al. 2021). Among these, organoids have the virtue of being relatively complex while also being composed of human cells, meaning that, if transcellular crossing is happening in BBB organoids, it is likely to also be important in clinical infections.

Unlike the 2D transwell model, where TEER can be measured to assess barrier integrity directly, the 3D system lacks such quantitative readouts. If C. neoformans secretes factors such as GXM or lytic enzymes that cause subtle, undetectable damage to the barrier, these effects would not be visible in the 3D structure. Such minor damage could lead to an apparent increase in nonspecific internalization. A brief acknowledgment of this limitation would make the discussion more balanced and complete.
While we think it is very unlikely, we agree with the reviewer that secreted fungal factors could potentially contribute to the partial loss of BBB integrity in 3D organoid. However, it does not follow that any loss BBB integrity is associated with nonspecific internalization, especially if the loss of integrity is too subtle to be easily detected. We note that, since *Cn* is comparable to mammalian cells in terms of size (relative to bacteria), any change that made barriers leaky enough to permit *Cn* between cells should be visible through normal light microscopy.

Previous studies in the transwell have demonstrated that crossing can occur without significant decrease in TEER (Chang et al. 2004). While it's not feasible to measure TEERs in organoid, prior studies have shown that in vitro BBB models developed from coculturing of BMECs with both pericytes and astrocytes increase the robustness of the barrier rather than weaken it (Urich, 2013). Thus, we believe that non-specific crossing of Cn is not a significant contributing factor to the data analysis because the organoid model was designed to work better than the 2D transwell model, which we had demonstrated to be an effective in vitro model of the BBB (Vu, 2009). In addition, the observation that *Cn* had a defect in crossing the *EphA2* KO organoids is consistent with the fact that nonspecific crossing of *Cn* was not significant (otherwise, we would not be able to observe the difference in *Cn* crossing between *EphA2* KO organoids and EphA2+ organoids).

In page 17, line 7, the Western blot section states that a C. neoformans lysate was prepared, yet the figure appears to analyze endothelial-cell lysate. The authors should clarify which lysate was actually used and ensure consistency between the Methods and Results descriptions.
This has been corrected.

Reviewer #4 (Remarks to the Author):

I co-reviewed this manuscript with one of the reviewers who provided the listed reports. This is part of the Communications Biology initiative to facilitate training in peer review and to provide appropriate recognition for Early Career Researchers who co-review manuscripts.
Thank you! We appreciate your service!
