# Supplementary material for: Macropinocytosis mediates neurotropism of Cryptococcus neoformans in a human organoid model of the blood-brain barrier
Source: Commun Biol. 2026 Jul 7;9:1430. doi: 10.1038/s42003-026-10597-y (PMC13720050; doi:10.1038/s42003-026-10597-y)
Supplement: Supplementary file 2 [file 42003_2026_10597_MOESM2_ESM.pdf]

nature portfolio

Corresponding author(s): Angie Gelli

Last updated by author(s): Oct 26, 2025

Reporting Summary

Nature Portfolio wishes to improve the reproducibility of the work that we publish. This form provides structure for consistency and transparency in reporting. For further information on Nature Portfolio policies, see our Editorial Policies and the Editorial Policy Checklist.

Statistics

For all statistical analyses, confirm that the following items are present in the figure legend, table legend, main text, or Methods section.

n/a	Confirmed	
☐	☒	The exact sample size (*n*) for each experimental group/condition, given as a discrete number and unit of measurement
☐	☒	A statement on whether measurements were taken from distinct samples or whether the same sample was measured repeatedly
☐	☒	The statistical test(s) used AND whether they are one- or two-sided<br>*Only common tests should be described solely by name; describe more complex techniques in the Methods section.*
☒	☐	A description of all covariates tested
☒	☐	A description of any assumptions or corrections, such as tests of normality and adjustment for multiple comparisons
☐	☒	A full description of the statistical parameters including central tendency (e.g. means) or other basic estimates (e.g. regression coefficient) AND variation (e.g. standard deviation) or associated estimates of uncertainty (e.g. confidence intervals)
☐	☒	For null hypothesis testing, the test statistic (e.g. *F*, *t*, *r*) with confidence intervals, effect sizes, degrees of freedom and *P* value noted<br>*Give P values as exact values whenever suitable.*
☒	☐	For Bayesian analysis, information on the choice of priors and Markov chain Monte Carlo settings
☒	☐	For hierarchical and complex designs, identification of the appropriate level for tests and full reporting of outcomes
☒	☐	Estimates of effect sizes (e.g. Cohen's *d*, Pearson's *r*), indicating how they were calculated

Our web collection on statistics for biologists contains articles on many of the points above.

Software and code

Policy information about availability of computer code

Data collection	Custom algorithms were not used for data collection.
Data analysis	Custom algorithms were not used for data analysis.

For manuscripts utilizing custom algorithms or software that are central to the research but not yet described in published literature, software must be made available to editors and reviewers. We strongly encourage code deposition in a community repository (e.g. GitHub). See the Nature Portfolio guidelines for submitting code & software for further information.

Data

Policy information about availability of data

All manuscripts must include a data availability statement. This statement should provide the following information, where applicable:

- Accession codes, unique identifiers, or web links for publicly available datasets
- A description of any restrictions on data availability
- For clinical datasets or third party data, please ensure that the statement adheres to our policy

The raw data is accessible in Dryad. DOI: 10.5061/dryad.w6m905r2p

Research involving human participants, their data, or biological material

Policy information about studies with human participants or human data. See also policy information about sex, gender (identity/presentation), and sexual orientation and race, ethnicity and racism.

Reporting on sex and gender	n/a
Reporting on race, ethnicity, or other socially relevant groupings	n/a
Population characteristics	n/a
Recruitment	n/a
Ethics oversight	n/a

Note that full information on the approval of the study protocol must also be provided in the manuscript.

Field-specific reporting

Please select the one below that is the best fit for your research. If you are not sure, read the appropriate sections before making your selection.

☒ Life sciences ☐ Behavioural & social sciences ☐ Ecological, evolutionary & environmental sciences

For a reference copy of the document with all sections, see nature.com/documents/nr-reporting-summary-flat.pdf

Life sciences study design

All studies must disclose on these points even when the disclosure is negative.

Sample size	In most cases final sample sizes were based on pilot studies. This approach allowed estimation of effect size and variance. We also performed power analysis to gage the appropriate sample size for sufficient power.
Data exclusions	Data were not excluded from the analyses performed in the present study.
Replication	We found that attempts at replication were successful. Typically at least three experimental replicates (technical and biological) were performed.
Randomization	Sample allocation was random.
Blinding	Generally, investigators were blinded to sample allocation for data analysis.

Reporting for specific materials, systems and methods

We require information from authors about some types of materials, experimental systems and methods used in many studies. Here, indicate whether each material, system or method listed is relevant to your study. If you are not sure if a list item applies to your research, read the appropriate section before selecting a response.

Materials & experimental systems

n/a	Involved in the study	
☐	☒	Antibodies
☐	☒	Eukaryotic cell lines
☒	☐	Palaeontology and archaeology
☒	☐	Animals and other organisms
☒	☐	Clinical data
☒	☐	Dual use research of concern
☒	☐	Plants

Methods

n/a	Involved in the study	
☒	☐	ChIP-seq
☒	☐	Flow cytometry
☒	☐	MRI-based neuroimaging

Antibodies

Antibodies used	A detailed list of antibodies used in this study, including type, vendor and catalogue number is provided in the extended files (or supplementary data).

Validation | Only commercial antibodies were used in the present study. We followed the published track record of immunoassays for the desired target. We also consulted published literature to identify the antibodies that succeeded, and the human protein atlas and/or the human gene database for information on target expression.

Eukaryotic cell lines

Policy information about cell lines and Sex and Gender in Research

Cell line source(s) | The immortalized cell line (hCMEC/D3 cells) was provided by Dr. B. Weksler (Wiell Cornell Medicine). Primary human brain endothelial cells, astrocytes and pericytes were purchased from a commercial vendor (ScienCell).

Authentication | Cell lines were not authenticated.

Mycoplasma contamination | We routinely test for mycoplasma contamination and we confirm that the immortalized cell lines and all primary cells were clear of mycoplasma.

Commonly misidentified lines (See ICLAC register) | n/a

Plants

Seed stocks | n/a

Novel plant genotypes | n/a

Authentication | n/a
