# Supplementary material for: Macropinocytosis mediates neurotropism of Cryptococcus neoformans in a human organoid model of the blood-brain barrier
Source: Commun Biol. 2026 Jul 7;9:1430. doi: 10.1038/s42003-026-10597-y (PMC13720050; doi:10.1038/s42003-026-10597-y)
Supplement: Supplementary file 3 [file 42003_2026_10597_MOESM3_ESM.pdf]

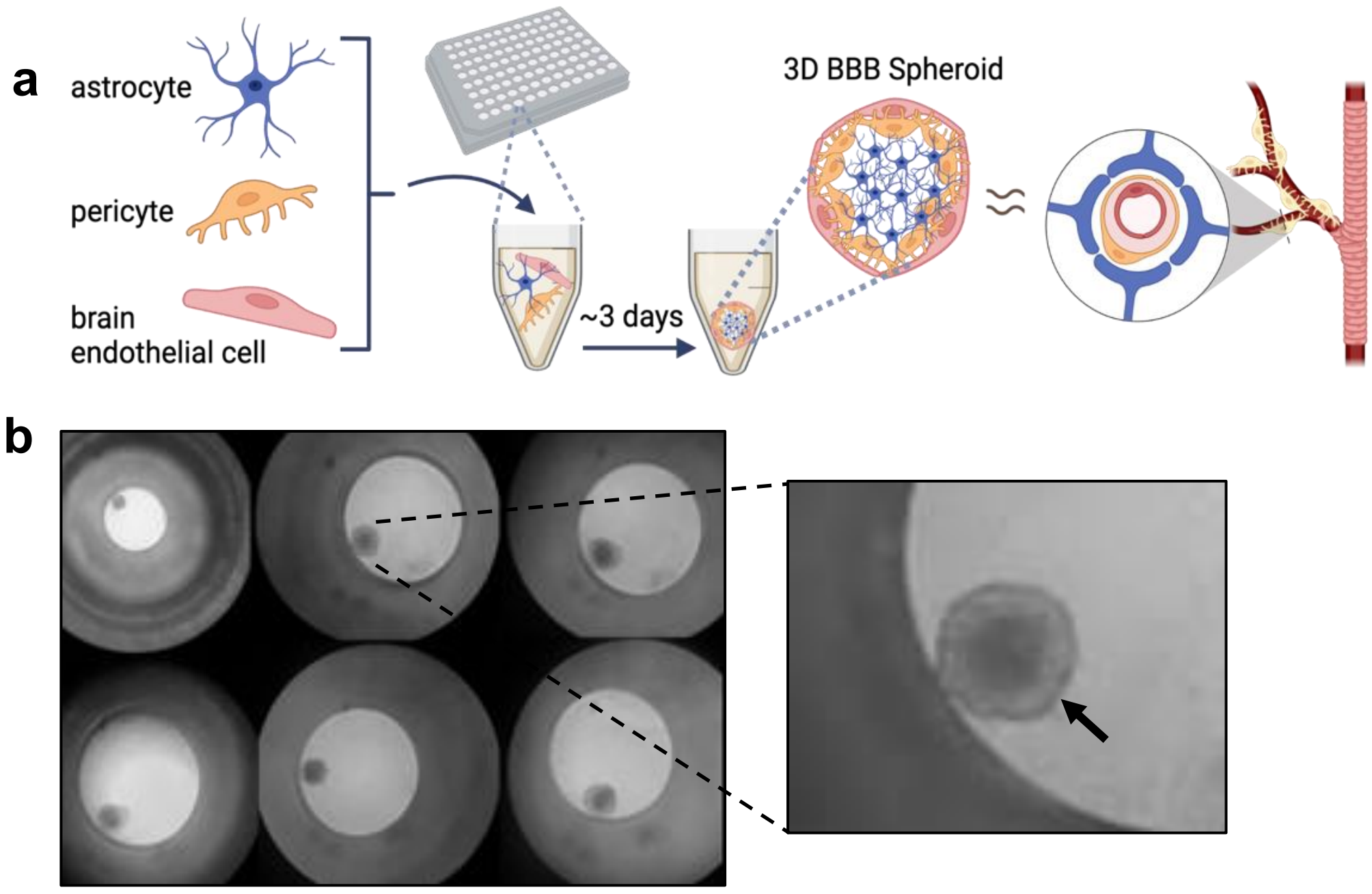

Supplementary Fig. 1. Schematics and images of 3D BBB organoid formation. **a** Organoids were formed from either primary human BMECs (passage < 6) or immortalized HCMEC/D3 cells (iBMECs, passage < 35), human primary pericytes (passage 4), and human astrocytes (passage 4). Primary cells were purchased from ScienCell (Carlsbad, California, USA). The organoids were formed by combining the three cell types in a 1:1:1 ratio, each at a concentration of 1×10^4 cells/mL. The combined cells were grown in 100 µL media in a 96-well plate. **b** Brightfield view of organoids formed at the bottom of flat-bottom 96-well plates with a transparent non-stick polymer coating. Organoids were relatively homogenous in size and shape.

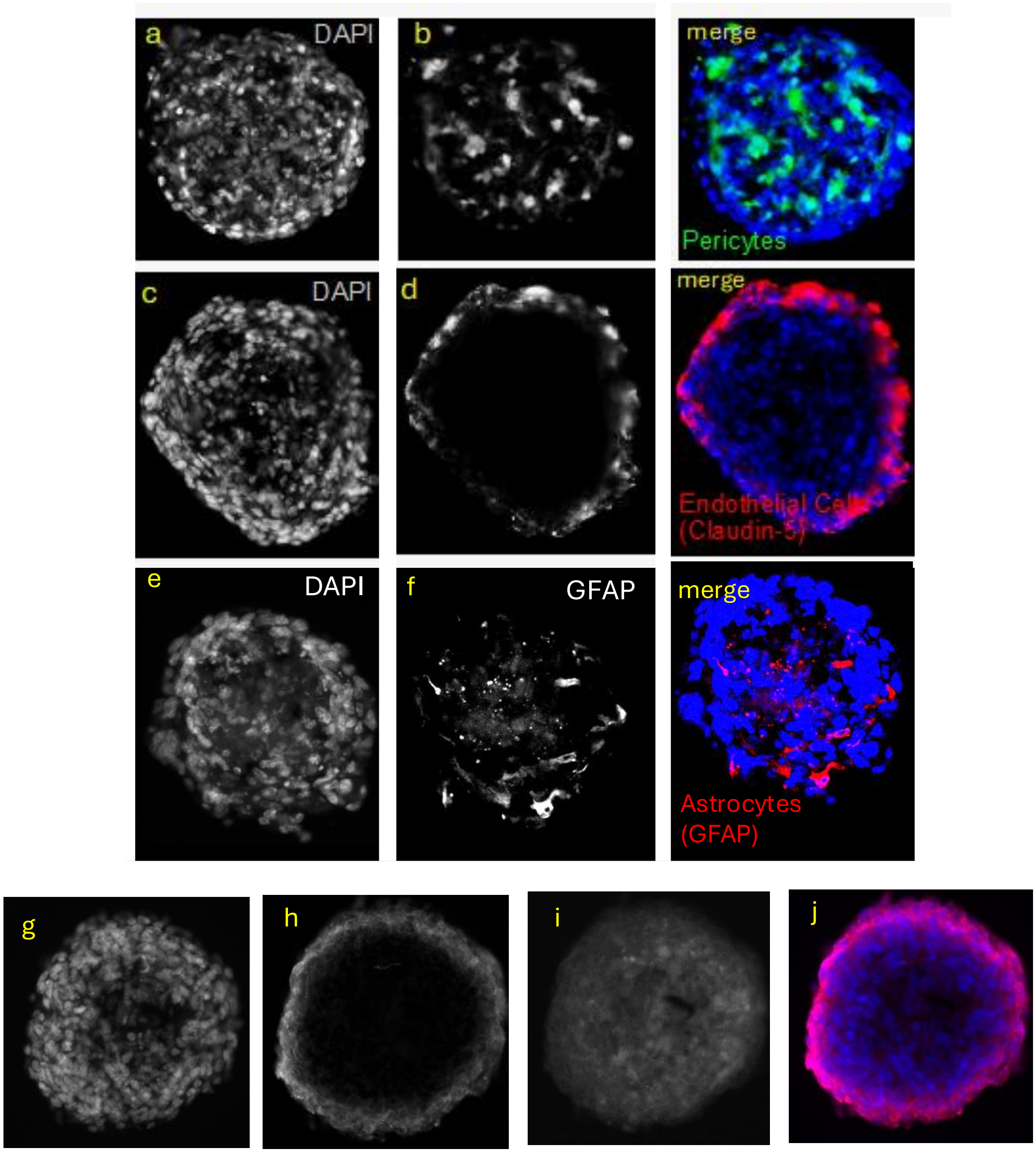

Supplementary Fig. 2. Organization of neurovascular cell types in human BBB organoids. First column **a**, **c**, **e** shows DAPI counterstaining. Second column **b**, **d**, **f** shows cell-type specific staining. Third column contains merged images from column 1 and column 2. Top row: pericytes were labeled in 2D culture with CMFDA (CellTracker Green, Invitrogen) and combined with endothelial cells and astrocytes to form organoids. **a** DAPI, **b** pericytes, merged image to the right. Middle row: Organoids were probed with **d** rabbit anti-Claudin5 (1:250) and goat - anti-rabbit TexasRed, merged image (middle row, right) with DAPI **c**. Bottom row: Astrocytes were visualized with GFAP (Invitrogen MA5-12023, 1:2000) **f** with DAPI counterstaining **e**, with merged images to the right. All images were acquired from 15µm thick cryosections of PFA-fixed sections made from 1-week old organoids. **g-j.** DAPI (**g**), claudin 5 (**h**), anti-*Cn* capsule (**i**) and **c**omposite (**j**) image of control (non-*Cn*-exposed) organoid section showing the expected peripheral localization of claudin 5 antibody (red) and the absence of non-specific anti-*Cn* capsule antibody (green channel). The image in **i** shows non-specific autofluorescence, with the gain adjusted to maximum settings, indicating that the anti-capsule (18b7) antibody does not bind to specific mammalian cell components. *Cn, Cryptococcus neoformans.*

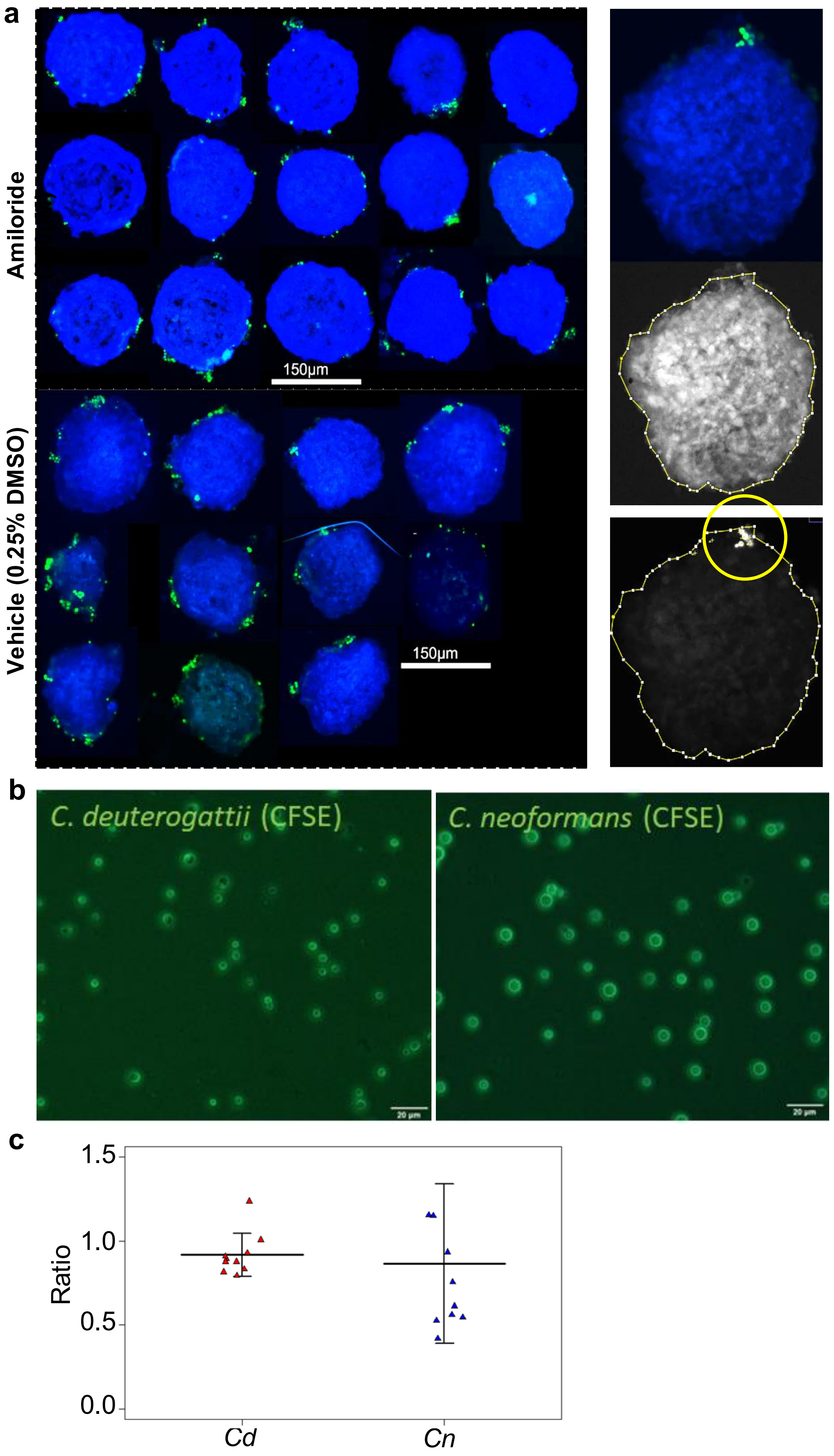

Supplementary Fig. 3. Organoid-based assays to examine the impact of AML treatment on *Cn* BBB-crossing/internalization. a Representative images showing organoids (blue, DAPI) incubated for 24 h with *Cn* (green, CFSE) in media containing either 250 µM amiloride (AML) (top-left) or an equivalent concentration of vehicle control (bottom left). Representative images from the analysis pipeline, with the "input" merged image at top right, border demarked around organoids in the DAPI channel (right middle panel), and this border applied in the CFSE channel to quantify crossing/internalization of fungal cells (bottom right panel, yellow circle). **b, c** Representative fields of view from a control experiment comparing CFSE-stained *Cn* (**b**, right) and *Cd* (**b**, left). Cells were stained with CFSE and incubated under mammalian tissue culture conditions. The ratio of stained (FITC channel) to total cells (transmitted light), as determined by an ImageJ script, does not differ between *Cn* and *Cd* (**c**). Differences in crossing between *Cn* and *Cd* are therefore not attributable to differences in affinity for CFSE. In a few cases, the script undercounted cells in the transmitted light channel, leading to ratios greater than one. *Cd, Cryptococcus deuterogattii; Cn, Cryptococcus neoformans.*

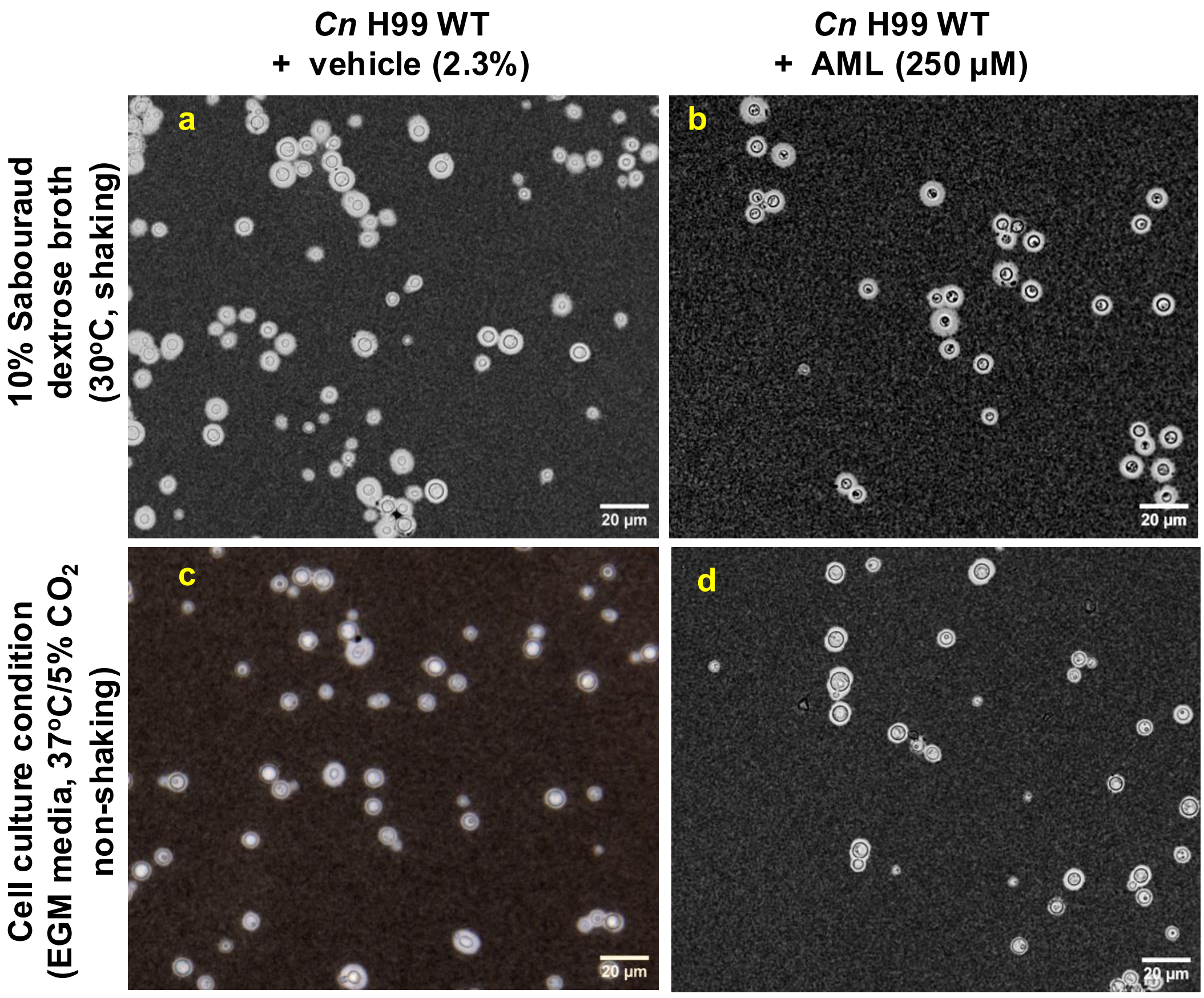

Supplementary Fig. 4. Amiloride (AML) treatment did not alter capsule expression of the *Cn* H99 wild-type (WT) strain. (**a, b)** To induce capsule, the *Cn* H99 WT strain was grown to stationary phase under either the capsule-inducing condition (10% Sabouraud dextrose broth, 30ºC, shaking) or mammalian growth condition (cell culture condition at 37ºC, 5% CO_2 in EGM media), respectively. Vehicle (DMSO) control (**a, c**) or 250 μM AML (**b, d**) were coincubated with the *Cn* H99 WT strain at the start of the assay. Capsules of cells were visualized using India ink (1μL ink + 3 μL of cells) under a brightfield microscope.

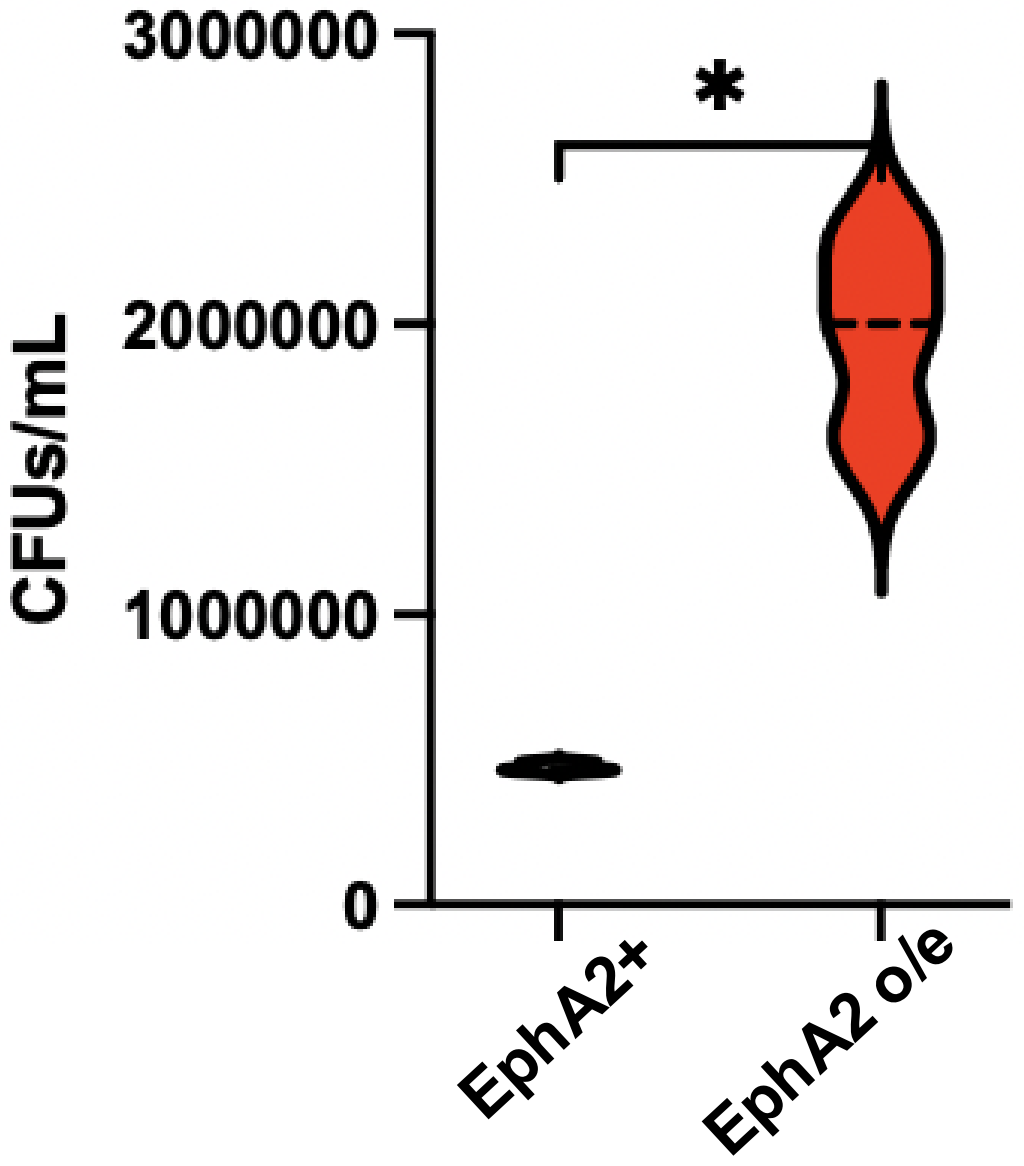

Supplementary Fig. 5. Overexpression of EphA2 in brain endothelial cells increases migration of *Cn* across the brain endothelium in an *in vitro* transwell model of the BBB. Transcytosis assay shows a significant increase in the movement of *Cn* across endothelial cells when brain endothelial cells (iBMECs) overexpress EphA2. EphA2+ iBMECs (wild type) and iBMECs transduced with EphA2 *via* lentivirus were exposed to *Cn* (MOI of 10) that was added to the apical side of the transwell. Following 12 h exposure, fungal cells were collected from the abluminal side of each transwell (well below transwell) and plated onto agar plates for CFU (colony forming units) determination. Each data point represents the fungal count from an individual well (n=3). Statistical analysis was done using a two-tailed, unpaired t-test with Welch correction ($p = 0.0178$, t= 7.358, df= 2.008)

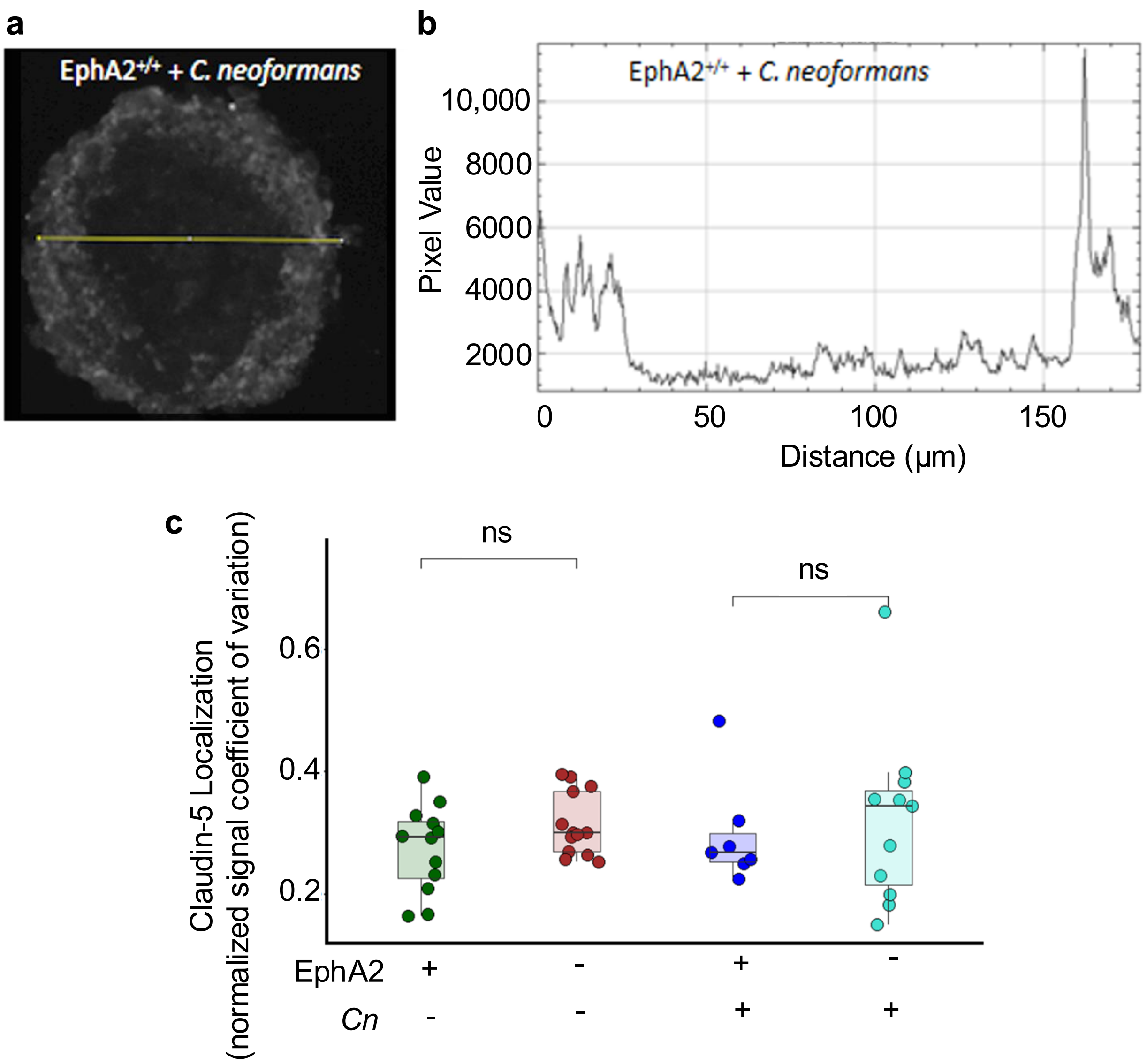

Supplementary Fig. 6. Claudin 5 expression in *EphA2 KO* organoids exposed to *Cn* a) Claudin5 signal (Cy5 channel) in cross-sections of BBB organoids. Sections were probed with rabbit anti-claudin 5 (1:1000, ab15106, Abcam) primary antibodies and goat anti-rabbit Cy5 (1:1000, ab6565, Abcam) secondary antibodies. **b)** Histograms corresponding to the pixel intensities measured across the diameter of organoids, prior to normalization and downstream analysis. **c)** Claudin 5 localization in organoids from *EphA2+* or *EphA2 KO* endothelial cells in the presence and absence of *Cn* (24 h coincubation). To create a metric of claudin5 localization within organoids, pixel intensity in the claudin5 (Cy5) channel across the diameter of organoid sections was acquired in ImageJ using the Plot Profile tool, normalized to the maximum value, and the coefficient of variation for each normalized vector was then taken. Points represent the average coefficient of variation across multiple sections for individual organoids (n=6). Data drawn from three independent biological replicates. There is no significant difference between *EphA2+* and *EphA2 KO* organoids. *Cn, Cryptococcus neoformans.*

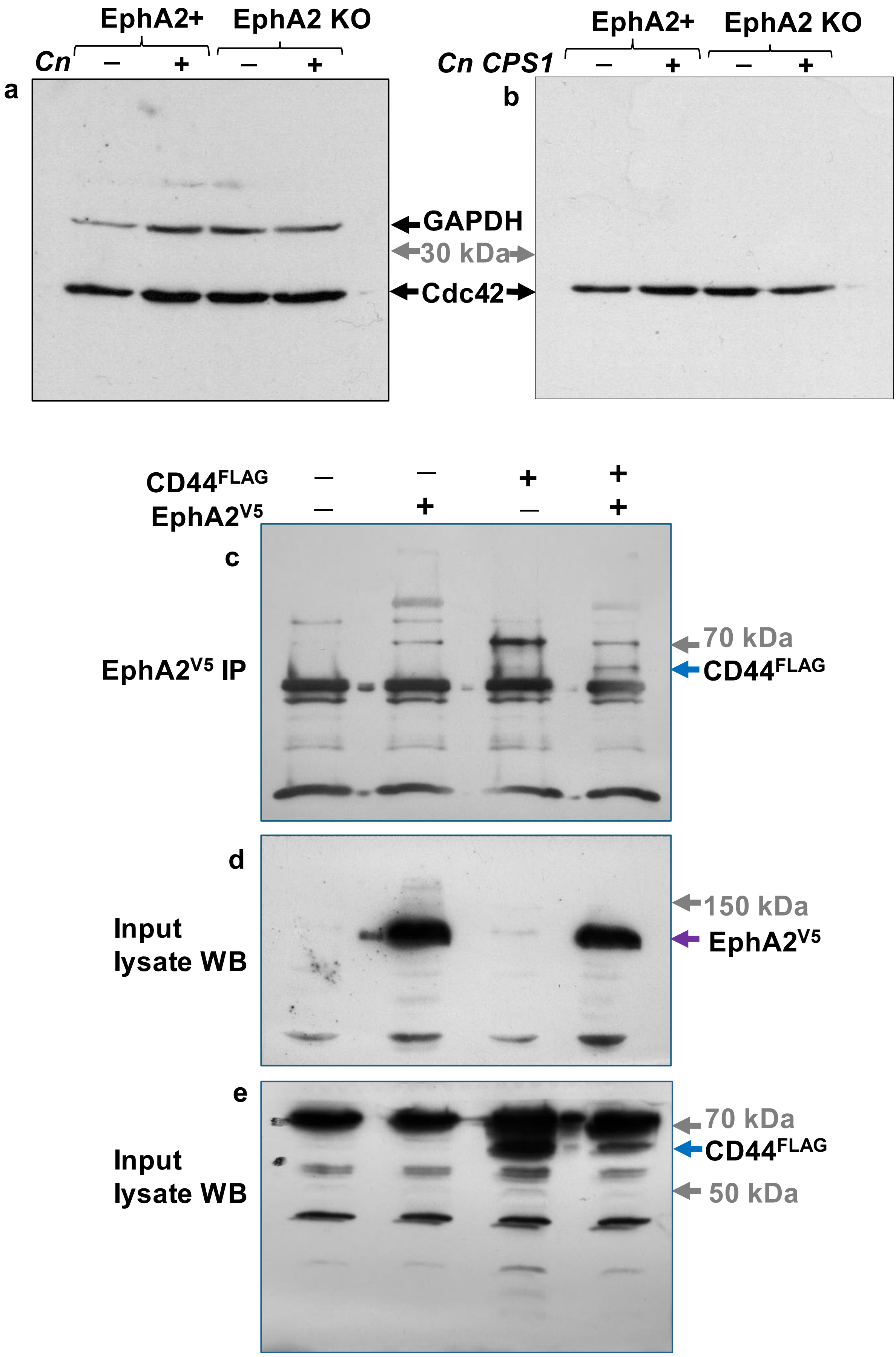

Supplementary Fig. 7. Immunoblots of SDS protein gels from the G-LISA co-immunoprecipitation (co-IP) studies. a, b, Immunoblots validate expression of Cdc42 (~21 kDa) in cell lysates used in G-LISA assays. **c, d, e,** Immunoblots detect expression and interaction of $EphA2^{V5}$ and $CD44^{FLAG}$. HEK293T cells transiently transfected with $EphA2^{V5}$ and/or $CD44^{FLAG}$ for 48 h were exposed to *Cn* for 2 h then immediately lysed in RIPA buffer supplemented with a protease (plus phosphatase) inhibitor cocktail. For co-immunoprecipitation studies, cell lysates were incubated with anti-V5 antibody for 2 h on ice followed by Dyna-Green Protein A/G magnetic beads that were subsequently thoroughly washed, added to Laemmli buffer and denatured proteins were separated by SDS PAGE electrophoresis and transferred to nitrocellulose membranes. Membranes showing co-IP or input lysates were incubated with mouse primary antibodies (1:800 dilution) of either anti-FLAG (top and lower panel) or anti-V5 (middle panel) followed by secondary antibodies (1:10,000 IgG H&L HRP). Proteins were visualized with enhanced chemiluminescence (ECL) reaction. WB, western blots.

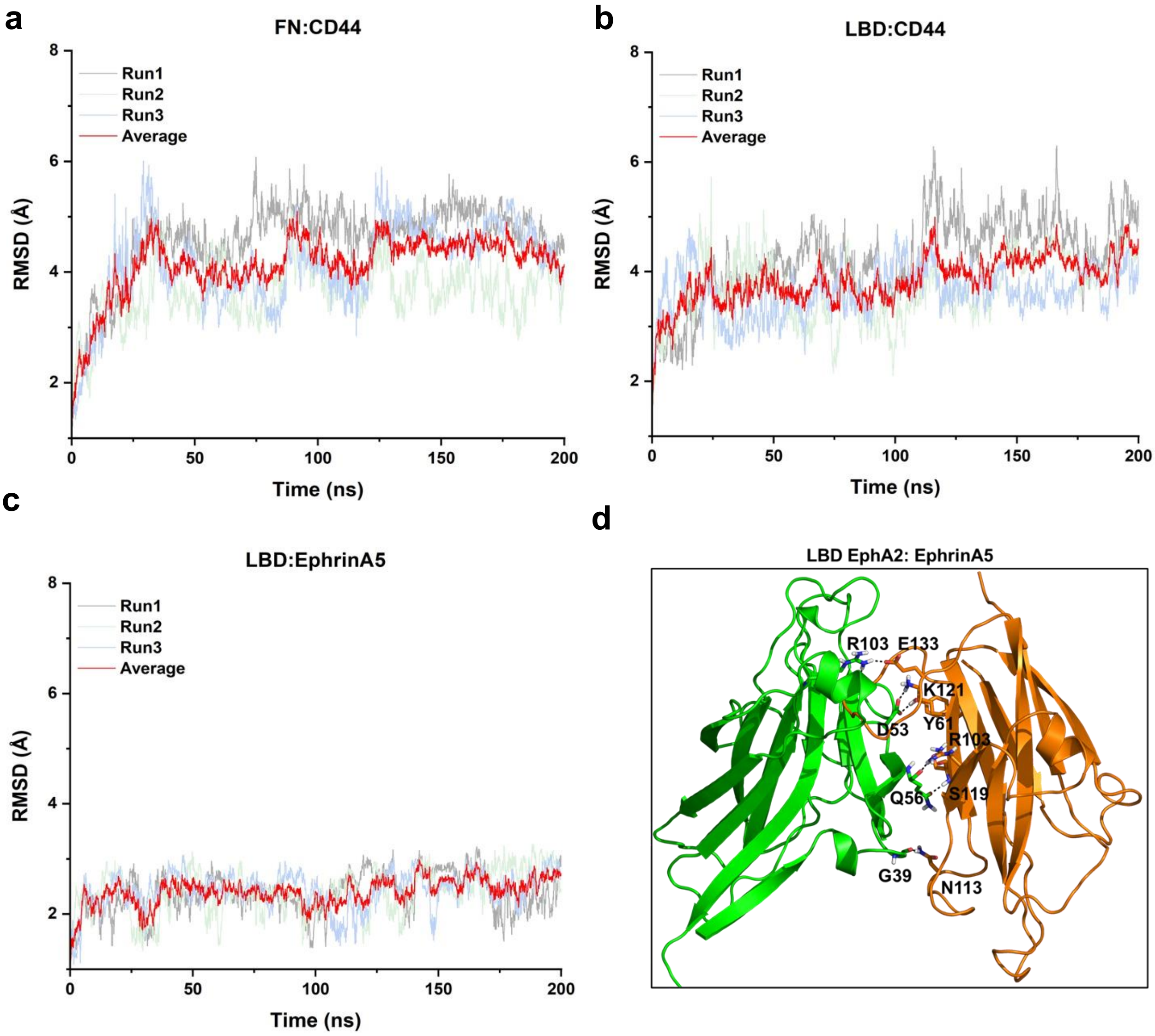

Supplementary Fig. 8. Structural stability and binding conformations of EphA2 complexes. **a–c** Backbone RMSD profiles of the FN: CD44, LBD: CD44, and LBD: ephrinA5 complexes over the course of molecular dynamics simulations. Data from all replica trajectories as well as averaged RMSD values are shown. **d** Representative binding conformation of the LBD: ephrinA5 complex obtained from clustering of all replica trajectories. EphA2 is depicted in green and ephrinA5 in orange cartoon representation. For clarity, only the major interfacial hydrogen bonds are displayed.

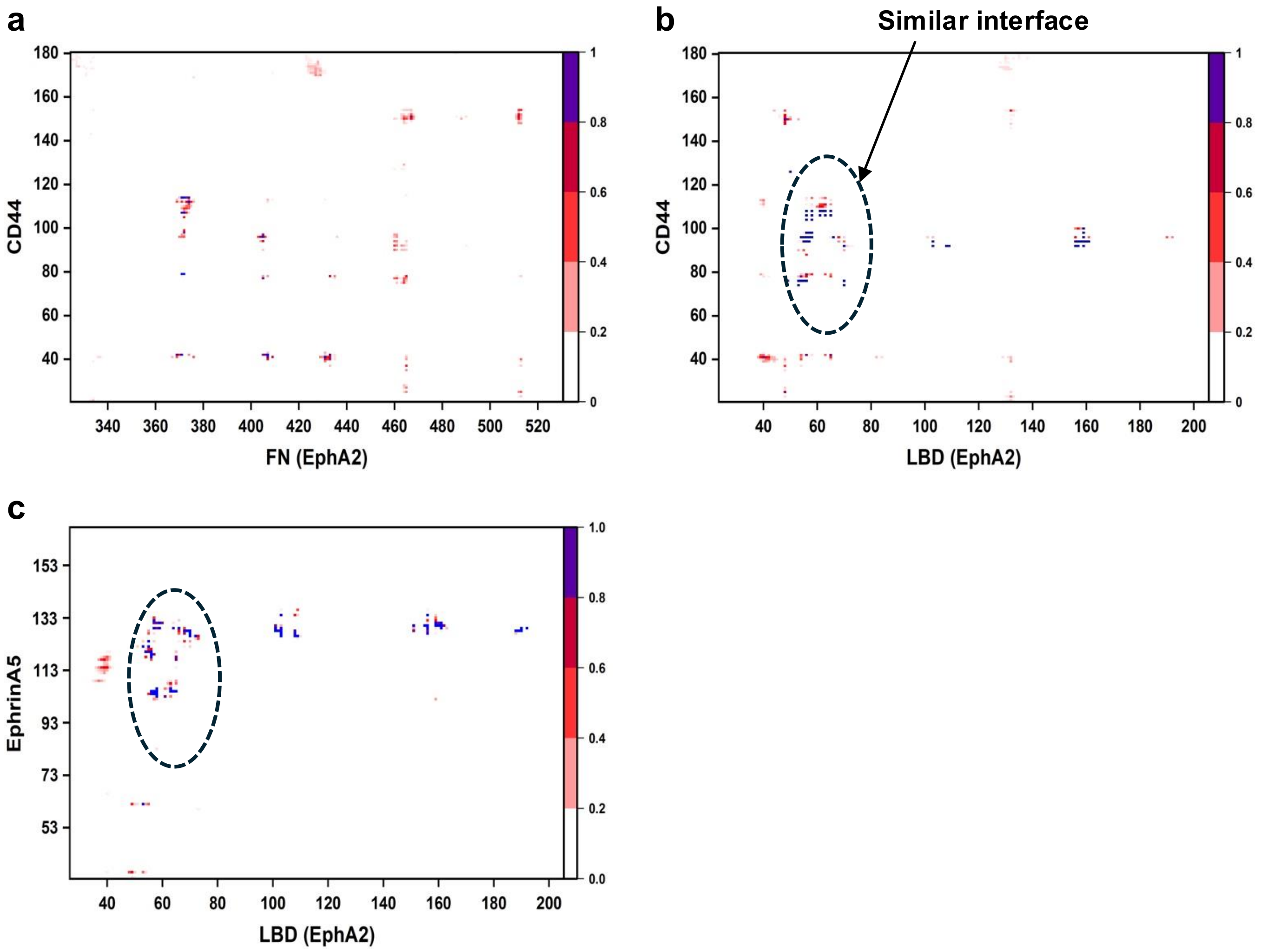

Supplementary Fig. 9. Intermolecular contact interfaces of EphA2 complexes. (**a–c**) Contact maps of the FN: CD44, LBD: CD44, and LBD: ephrinA5 complexes obtained from molecular dynamics simulations. Contacts were calculated using a 5 Å cutoff. The color scale (white to red to blue) represents the fractional occupancy of each contact, ranging from 0 (no contact) to 1 (persistent contact). Data from all replica trajectories were included in the analysis. Notably, CD44 and ephrinA5 engage overlapping interface regions within the EphA2 LBD.
